# Physical, mechanical, and microstructural characterisation of tungsten carbide-based polymeric composites for radiation shielding application

**DOI:** 10.1038/s41598-023-49842-3

**Published:** 2024-01-16

**Authors:** Nadin Jamal Abualroos, Mohd Idzat Idris, Haidi Ibrahim, Muhammad Izzat Kamaruzaman, Rafidah Zainon

**Affiliations:** 1 Department of Biomedical Imaging, Advanced Medical and Dental Institute, SAINS@BERTAM, Universiti Sains Malaysia, 13200 Kepala Batas, Pulau Pinang Malaysia; 2 Health Sciences Centre, School of Medicine, University College Dublin, Belfield, Dublin 4, Ireland; 3 Nuclear Technology Research Centre, Department of Applied Physics, Faculty of Science and Technology, Universiti Kebangsaan Malaysia, UKM, 43600 Bangi, Selangor Malaysia; 4 School of Electrical and Electronic Engineering, USM Engineering Campus, Universiti Sains Malaysia, 14300 Nibong Tebal, Pulau Pinang Malaysia; 5 Chembio Technology Sdn Bhd, 19, Persiaran Seksyen 4/3, Bandar Putra Bertam, 13200 Kepala Batas, Pulau Pinang Malaysia

**Keywords:** Materials science, Physics

## Abstract

Polymeric based composites have gained considerable attention as potential candidates for advanced radiation shielding applications due to their unique combination of high-density, radiation attenuation properties and improved mechanical strength. This study focuses on the comprehensive characterisation of polymeric based composites for radiation shielding applications. The objective of this study was to evaluate the physical, mechanical and microstructural properties of tungsten carbide-based epoxy resin and tungsten carbide cobalt-based epoxy resin for its efficiency in shielding against gamma-rays ranging from 0.6 up to 1.33 MeV. Polymeric composites with different weight percentages of epoxy resin (40 wt%, 35 wt%, 30 wt%, 25 wt%, 20 wt%, 15 wt% and 10 wt%) were fabricated, investigated and compared to conventional lead shield. The attenuation of the composites was performed using NaI (Tl) gamma-ray spectrometer to investigate the linear and mass attenuation coefficients, half value layer, and mean free path. High filler loadings into epoxy resin matrix (90% filler/10% epoxy) exhibited excellent gamma shielding properties. Mechanical properties, such as hardness were examined to assess the structural integrity and durability of the composites under various conditions. The fabricated composites showed a good resistance, the maximum hardness was attributed to composites with small thickness. The high loading of fillers in the epoxy matrix improved the microhardness of the composites. The distribution of the filler powder within the epoxy matrix was investigated using FESEM/EDX. The results revealed the successful incorporation of tungsten carbide and cobalt particles into the polymer matrix, leading to increased composite density and enhanced radiation attenuation. The unique combination of high-density, radiation attenuation, and improved mechanical properties positions polymeric based composites as promising candidates for radiation protection field.

## Introduction

Lead has been utilised as a radiation shielding material in a variety of applications such as industrial shielding, diagnostic imaging, radiation therapy and nuclear shielding, due to its high density, high atomic number, and high linear and mass attenuation coefficients for X-rays and gamma radiation^1,2^. However, due to its hazardous effects on humans and the environment, the use of lead has been restricted in various equipment and applications according to the Restriction of Hazardous Substances (RoHS) directive of the European Union^3–5^.

When determining the most suitable type of radiation shielding, factors such as cost, weight, and chemical and physical durability are taken into consideration. It is important to note that materials with a high density and atomic number offer greater attenuation than those with lower atomic numbers^6^. However, the use of heavy metals like lead can have long-term negative effects on both human health and the environment. This has led to a growing interest in the development of non-toxic, lightweight, flexible, and cost-effective materials for radiation shielding, as a substitute for lead^7^.

Composite materials are composed of two or more components, each of which possesses unique physical and chemical properties^8^. Non-toxic polymer composites are ideal for radiation shielding due to their light weight and environmental friendly qualities^9,10^. It possesses unique optical and electrical qualities, as well as reasonable costs, high flexibility, and outstanding mechanical strength. Polymer composites can be doped with high atomic number elements in addition to lead to protect against gamma ray. Despite having a lower effective density than lead, these composites can provide adequate protection against gamma radiation^11^.

Tungsten alloys and tungsten-based materials are frequently used in radiation shielding applications because of their high atomic number, density, and hardness^12^. These compounds have effects that are remarkably comparable to those of the conventional radiation shielding materials^13^. Tungsten carbide is also known to have shielding properties comparable to those of lead. Furthermore, tungsten, boron carbide, and tungsten carbide used as fillers in a metal-polymer composites based on ultra-high molecular weight polyethylene to shield against gamma radiation^14^.

Epoxy is a thermoset polymer that is commonly used in the nuclear industry because of its light weight, ease of processing, excellent mechanical qualities, radiation, corrosion, and chemical stability. Furthermore, it can be made to fit into any mould shape^15^. Epoxy composites with high atomic number fillers have been developed as low-energy gamma and X-ray shielding materials^16,17^. Additionally, several investigations on the radiation stability of epoxy resins used in various applications have been studied^18,19^.

The mechanical and chemical properties of epoxy resin composites are excellent. It has been demonstrated in the literature that heavy metal powders such as tungsten with varying weight percent can enhance the shielding capacity of epoxy resin^6^. Giménez et al.^20^ has combined tungsten carbide sintered pellets and lead as filling material for optimised radiation shielding design for small and medium reactors. In addition, Chang et al.^21^ conducted a study on epoxy composites incorporating 6 μm tungsten, examining their radiation shielding properties with two activities of Co-60. The doping of tungsten improved the shielding and mechanical properties of the tungsten-based epoxy composites. Additionally, fiber-reinforced epoxy resin composites are regarded an attractive material for nuclear research since they retain exceptional fatigue strength, thermal conductivities, and extraordinary dimensional and thermal stabilities even after gamma ray irradiation^22^. In this study, we investigated the efficacy of polymeric composites containing micro-sized tungsten carbide particles and nano-sized tungsten carbide cobalt particles for radiation shielding. This study provides a comprehensive analysis of physical, mechanical, and microstructural characteristics of tungsten carbide-based polymeric composites for in shielding against gamma-rays ranging from 0.6 up to 1.33 MeV.

## Methodology

The experimental work of this study involved several steps including preparation of the samples, and the equipment used for physical, chemical, mechanical, and radiation shielding characterisation of the fabricated samples.

### Materials

**Figure 1:**
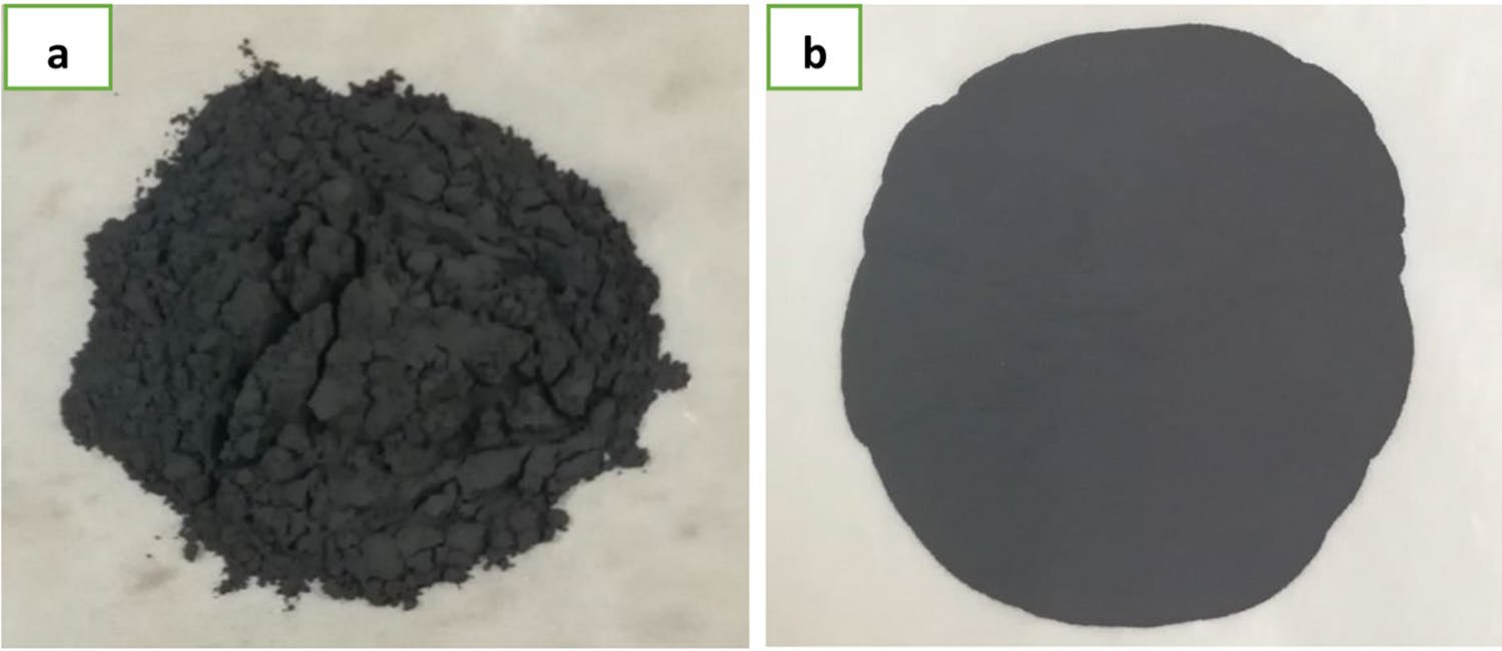
(**a**) Tungsten carbide powder and (**b**) tungsten carbide cobalt powder.

The tungsten carbide powder purchased from SAT Nanotechnology Materials Co Ltd—Guangdong, China was 99.95% pure and had an aerodynamic particle sizer (APS) of 1–2 μm. Tungsten carbide is fine powder made of tungsten and carbon as shown in Fig. 1a. Tungsten carbide mixed with epoxy resin at different weight percentage to study the best composite combination that has the best radiation shielding qualities among all fabricated composites.

Tungsten carbide cobalt powder purchased from SAT nanotechnology materials CO Ltd—Guangdong, China of 99.9% purity and aerodynamic particle sizer APS: 100 nm. Tungsten carbide cobalt is carbide that made of tungsten carbide and cobalt. Tungsten carbide cobalt powder is shown in Fig. 1b. Tungsten carbide cobalt mixed with epoxy resin polymer at different weight percentages to ensure the best weight percentage of combination between the powder and the epoxy through evaluating the shielding properties of all different samples. General properties of tungsten carbide and tungsten carbide cobalt powder are summarised in Table 1.

**Table 1:** General properties of tungsten carbide and tungsten carbide cobalt powders.

General properties	Tungsten carbide	Tungsten carbide cobalt
Particle (APS)	1–2 μm	60–100 nm
Form	Powder	Powder
Molecular formula	WC	WC–Co
Purity	99.95%	99.9%
Density	15.63 g/cm^3^	14.95 g/cm^3^
Crystal shape	Hexagonal	Spherical
Color	Dark grey	Dark grey
Odor	Odorless	Odorless
Solubility in water	Insoluble	Insoluble
Melting point	2870 °C	2867 °C
Boiling point	6000 °C	6000 °C

Table 2 summarised the physical, chemical, and mechanical properties of epoxy resin and hardener type (E-110I/H-9) that purchased from Pan Asel Chemicals (M) Sdn Bhd, Kuala Lumpur—Malaysia.

**Table 2:** Physical, chemical, and mechanical properties of E-110I/H-9 epoxy resin.

Properties	Resin (E-110I)	Hardener (H-9)
Appearance	Clear liquid	Clear liquid
Viscosity (cps)	3500–4500	100–150
Mixing ratio (by weight)	2	1
Density (g/cm^3^)	1.1	0.9
Pot life	60 min	60 min
Shelf life	6 months	6 months
Cure condition	14–16 h (Room temperature) 70 °C: 70–80 min	14–16 h (Room temperature) 70 °C: 70–80 min
Compression strength (kg/cm^2^)	728	718
Flexural strength (kg/cm^2^)	297	297
Tensile strength (kg/cm^2^)	136	136
Flash point	> 300 °C	> 150 °C

### Samples preparation

Tungsten carbide-based epoxy resin composites were prepared for field emission scanning electron microscope (FESEM) and energy dispersive X-ray (EDX), FTIR, gamma spectrometry, and mechanical properties analyses. To determine the optimal weight percentage of powder filler and epoxy for effective shielding against gamma radiation, various composites of powder and epoxy resin were prepared in different weight percentages as detailed in Table 3 during the initial stage of sample preparation. After identifying the optimal combination of materials, bricks with thicknesses of 0.7 cm and 1.4 cm were produced and utilised in a nuclear medicine unit.

**Table 3:** A list of prepared samples with different weight percentage of powder filling and epoxy resin.

Sample	Tungsten carbide (wt%)	Epoxy resin (wt%)	Sample	Tungsten carbide cobalt (wt%)	Epoxy resin (wt%)
WCE1	60	40	WCoE1	60	40
WCE2	65	35	WCoE2	65	35
WCE3	70	30	WCoE3	70	30
WCE4	75	25	WCoE4	75	25
WCE5	80	20	WCoE5	80	20
WCE6	85	15	WCoE6	85	15
WCE7	90	10	WCoE7	90	10

An open mould casting technique was used to prepare the polymeric composites. Tungsten carbide-based epoxy resin composites and tungsten carbide cobalt-based epoxy resin composites were fabricated with two different thicknesses 0.7 cm and 1.4 cm, respectively. The preparation of tungsten carbide-based epoxy resin and tungsten carbide cobalt-based epoxy resin composites were carried out by loading filler powder with different weight percentages to epoxy resin and hardener. The mixing process of epoxy powder composite was performed by using electric mixture for 20 min.

Then, a magnetic stirrer was used to ensure uniform dispersion of the powder in the epoxy resin matrix (460–800 rpm). Weighting of powders and epoxy resin was carried out by using A&D weighting GR-200 lab analytical balance. The stirring process was performed slowly to reduce the formation of air bubbles within the mixture of samples. After achieving a thorough mixture, the composite was poured into silicone moulds and with thicknesses of 0.7 cm and 1.4 cm. The mixture was left at room temperature for 24 h. The mass ratio of epoxy resin to hardener is 2:1.

**Figure 2:**
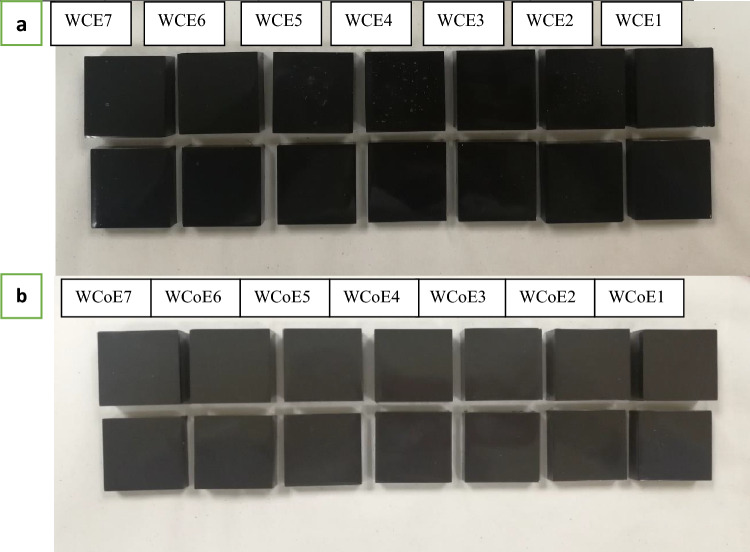
(**a**) Tungsten carbide-based epoxy composites, with the top one having a thickness of 1.4 cm and the bottom one having a thickness of 0.7 cm, and (**b**) Tungsten carbide-based epoxy resin composites with the upper one having a thickness of 1.4 cm and the lower one having a thickness of 0.7 cm.

The list of prepared samples with different weight percentages of tungsten carbide-based epoxy resin and tungsten carbide cobalt-based epoxy resin is shown in Table 3 and Fig. 2. A combination of tungsten carbide and epoxy resin was denoted as WCE, while a combination of tungsten carbide cobalt and epoxy resin was denoted as WCoE in which WC represents tungsten carbide, WCo represents tungsten carbide cobalt, E represents epoxy resin, and X represents the number of the sample.

### Density measurement

The density of the fabricated samples was determined during the fabrication process. Density of the fabricated tungsten carbide based-epoxy resin composites was defined by the Eq. (1)^23^.

1 $${\rho }_{m}=\frac{100}{\frac{{c}_{\omega }}{{\rho }_{s}}+\frac{100-{c}_{w}}{{\rho }_{l}}}$$

where ${\rho }_{m}$ is the density of the mixture, ${c}_{\omega }$ is the concentration by weight in percent, ${\rho }_{l}$ is the density of epoxy resin, and ${\rho }_{s}$ is the density of filler powder.

### Field emission scanning electron microscope (FESEM)/ Energy dispersive X-ray (EDX)

In this study, FESEM/EDX have been used to determine the effect of the powder and epoxy ratio on the properties and microstructure of the different fabricated composites. The morphological and microstructural properties of the fabricated tungsten carbide-based epoxy resin composites were investigated using Extreme High Resolution Field Emission Scanning Electron Microscope (XHR-FESEM) Model FEI Verios 460L, Science and Engineering Research Centre Lab, Engineering Campus, Universiti Sains Malaysia.

FESEM was employed to investigate the surface morphology of tungsten carbide powder and tungsten carbide cobalt powder, and to ensure the purity of the materials used. The elemental analysis was carried out by using EDX to determine the percentage of elements present in the samples. The samples were prepared to meet the specific requirements of the instrument. Square shape of 2 cm × 2 cm tungsten carbide-based epoxy resin composites samples was investigated by using XHR-FESEM.

Before being observed in FESEM, a Gatan precision etching coating system Model 682, Science and Engineering Research Centre Lab, Engineering Campus, Universiti Sains Malaysia. was utilised to apply a gold coating to all samples. Table 4 shows the fabricated samples with its molecular formula and type that were characterised by using FESEM/EDX analysis test. Four different weight percentages samples among all samples were chosen for FESEM analysis test. The list of tungsten carbide-based epoxy composites and tungsten carbide cobalt-based epoxy composites that evaluated by FESEM/EDX were listed in Table 5.

**Table 4:** The samples were characterised by using FESEM/EDX analysis.

Sample	Molecular formula	Type
Tungsten carbide	WC	Powder
Tungsten carbide cobalt	WC–Co	Powder
Epoxy resin and hardener	C_21_H_25_ClO_5_	Solid

**Table 5:** The selected tungsten carbide-based epoxy resin and tungsten carbide cobalt-based epoxy resin composites for FESEM/EDX analysis test.

Sample	Tungsten carbide (wt%)	Epoxy resin (wt%)	Sample	Tungsten carbide cobalt (wt%)	Epoxy resin (wt%)
WCE2	65	35	WCoE2	65	35
WCE4	75	25	WCoE4	75	25
WCE6	85	15	WCoE6	85	15
WCE7	90	10	WCoE7	90	10

### Fourier transform infrared spectroscopy (FTIR)

In this study, Fourier-transform infrared spectroscopy technique was used to compare the change of the chemical bonds in the epoxy matrix, and the effect of adding different powders to the epoxy resin base. The FTIR analysis was performed at Science and Engineering Research Centre Lab, Engineering Campus, USM. 400 Spotlight FTIR Imaging system integrated with Attenuated Total Reflection (ATR) model from PerkinElmer, USA for Mid-NIR infrared analysing.

In this study, the FTIR was used to determine the presence of the chemical functional group and to determine the changes in the molecular structures of the different composites. The Zinc Selenide detector is used to detect the transmission of spectra with scan ranging from 650 to 4000 cm^−1^. A resolution of 8 cm^−1^ was used in the scanning. The spectrum is processed using PerkinElmer's IR spectrum software version 10.3.2. Three different samples were examined by FTIR including epoxy resin sample, tungsten carbide-based epoxy resin and tungsten carbide cobalt-based epoxy resin samples. The infrared spectrum was divided into three main wavelength regions, the far, the mid, and the near infrared spectrums. The most widely used spectrum in the sample analysis was the mid infrared spectrum^24^.

### Vickers hardness test

**Figure 3:**
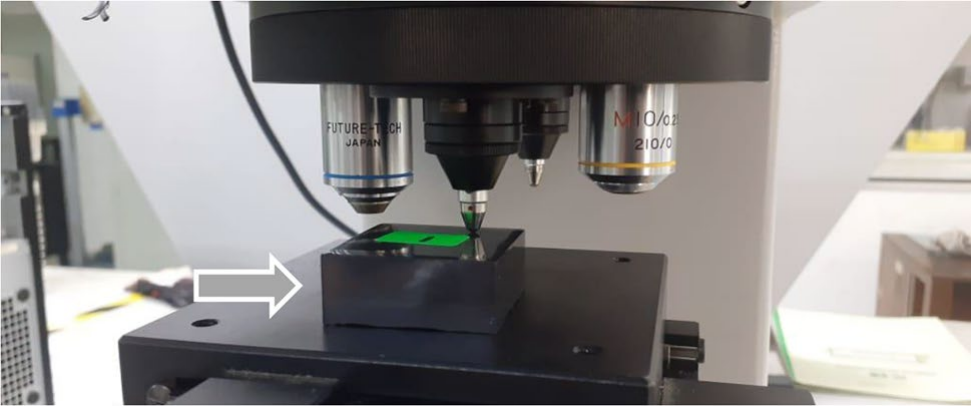
The fabricated sample was placed under hardness testing machine.

The fabricated samples' hardness was assessed using the Vickers hardness test in the micro hardness test load range. The hardness mechanical property of the fabricated samples was evaluated using Microhardness Tester (Model LM248AT – LECO Corporation Michigan – USA) at USM Physics Laboratory, School of Materials and Mineral Resources, Engineering Campus in Universiti Sains Malaysia as shown in Fig. 3. The equipment was connected to advanced colour touch panel display that used to easily access all operating parameters. The test load on the indenter of the microhardness tester was ranging from 1 to 2000 g force. In this study, 1 kg-force (kgf) of load was used for five seconds. The Vickers hardness values were calculated based on the dimensions of the indentation created by the testing machine, which was automatically measured. The test was performed based on ASTM E384-17 standards. For each sample, three points measurements were taken to get the mean hardness value. The composite hardness values were investigated because they are essential and clinically significant indicators of the effectiveness of condensable composite resins' curing processes.

### Gamma ray attenuation measurement

Gamma spectroscopy experiment conducted in gamma spectroscopy laboratory, at Nuclear Science Laboratory in School of Science and Technology, National University of Malaysia. In this experiment setup, sodium iodide (NaI) scintillation detector with channel analyser Brand and Model (Canberra/Genie 2K), high voltage power supply Model 3102D, amplifier, and dual counter timer were used to measure the transmission of gamma photons.

**Figure 4:**
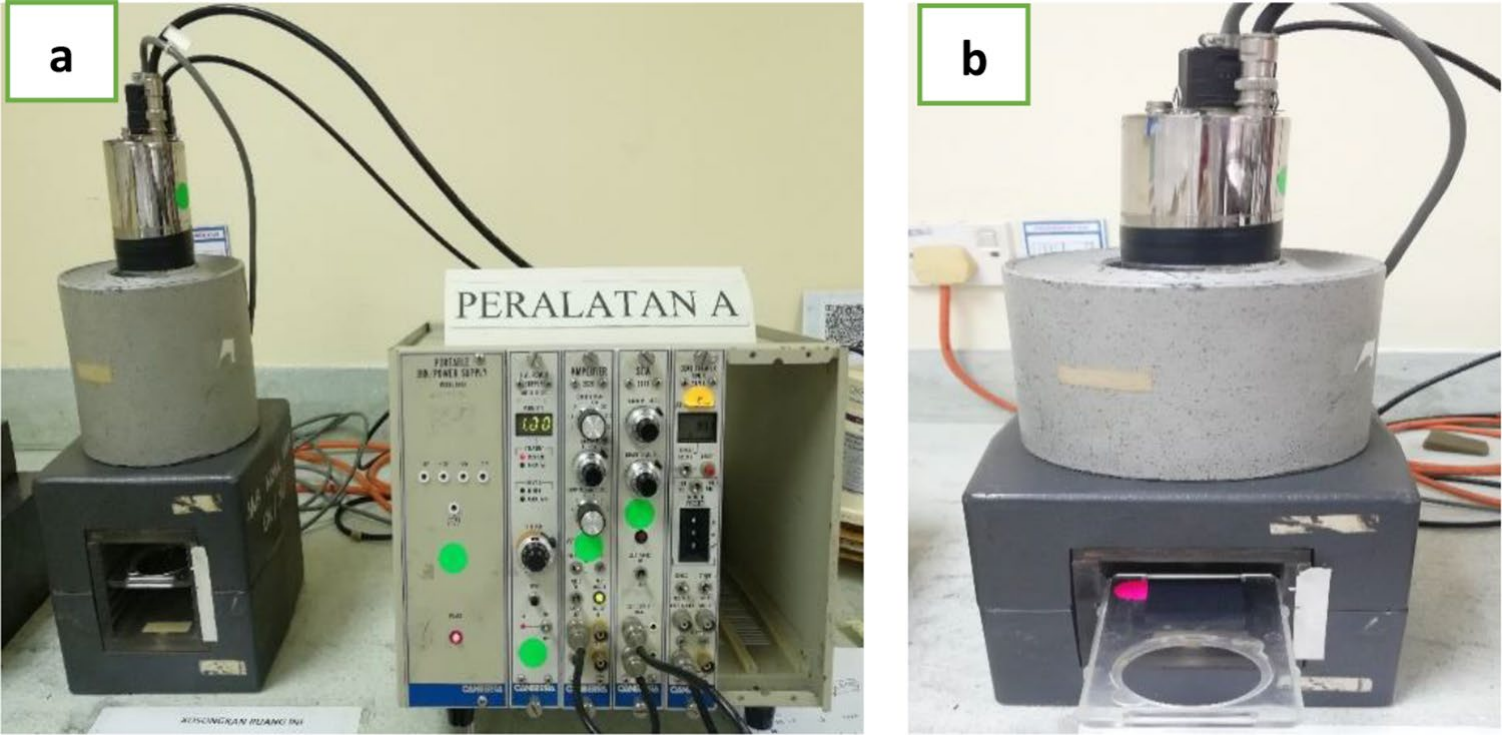
(**a**) The experimental setup for the attenuation measurements, and (**b**) Gamma ray spectroscopy experiment setup with sample placed between radiation source and detector.

In this study, Americium-241 (^241^Am), Barium-133 (^133^Ba), Sodium-22 (^22^Na), Cesium-137 (^137^Cs), and Cobalt-60 (^60^Co) were used as gamma photons sources to evaluate the radiation attenuation properties of the fabricated tungsten carbide based-epoxy resin composites. Those radiation sources were selected based on their respective gamma emission energies that covered the energy range of diagnostic and therapeutic field. Table 6 shows the physical and chemical properties of the selected radiation point sources. The experimental setup of the radiation attenuation measurements is shown in Fig. 4.

**Table 6:** Chemical and physical properties of the selected radiation point sources.

Properties	^241^Am	^133^Ba	^22^Na	^137^Cs	^60^Co
Atomic number	95	56	11	55	27
Density (g.cm^−3^ at 20°C)	12	3.51	0.7	1.93	8.86
Melting point (°C)	1176	727	97.794	28.44	1495
Half-life	432.2 Y	10.551 Y	2.6 Y	30 Y	5.26 Y
Gamma emission energy (keV)	60	80, 160, 223, 356	511	661	1173.2, 1332.5

The probability of gamma ray interaction with a material per unit path length is defined by linear attenuation coefficient (*µ*). According to lambert–Beer law, when a radiation beam incident on the matter, it will be attenuated. The attenuation of gamma radiation can be calculated based on Eq. (2):

2 $$I={I}_{0}{e}^{-\mu x}$$

where *I*_*o*_ denotes the initial photon intensity; and *I* is the photon intensity transmitted through sample with thickness *x*. The mass attenuation coefficient (${\mu }_{m}$) is a describes the average number of interactions that occurs between incident photons and matter. Determination of mass attenuation coefficient of the materials was performed by dividing the linear attenuation coefficient of the material by the density of the material according to Eq. (3):

3 $${\mu }_{m}=\frac{\mu }{\rho }$$

where $\rho$ is the density of the material.

The mean free path also known as relaxation time (*λ*) is the average distance between two successive interactions of photons in which the intensity of the incident photon beam is reduced by the factor 1/e. It can be calculated using the value of the linear attenuation coefficient as shown in Eq. (4).

4 $$\lambda =\frac{1}{\mu }$$

The half value layer (HVL) and tenth value layer (TVL) are terms that represent the thickness of an absorber that will reduce the gamma radiation to one half and one tenth of the initial intensity, respectively. These terms used basically to analyse the penetrating ability of gamma radiation through the proposed shielding materials. The HVL and TVL are important parameters, and they are given as shown in Eqs. (5) and (6):

5 $$HVL=\frac{\mathit{ln}2}{\mu }$$

6 $$TVL=\frac{\mathit{ln}10}{\mu }$$

The radiation protection efficiency (RPE) is an important parameter that gives information on the shielding ability of the proposed material. It can be determined as shown in Eq. (7):

7 $$RPE=\left(1-\frac{I}{{I}_{0}}\right)\times 100$$

## Results and discussion

### Density of the samples

The calculated density values for the fabricated samples with their filler loading percentages are listed in Table 7.

**Table 7:** Density of the fabricated WCE and WCoE samples.

Samples	Filler concentration of composites (%)	Density (g/cm^3^)	Samples	Filler concentration of composites (%)	Density (g/cm^3^)
WCE1	60	2.419	WCoE1	60	2.409
WCE2	65	2.705	WCoE2	65	2.691
WCE3	70	3.067	WCoE3	70	3.048
WCE4	75	3.542	WCoE4	75	3.515
WCE5	80	4.190	WCoE5	80	4.150
WCE6	85	5.129	WCoE6	85	5.064
WCE7	90	6.609	WCoE7	90	6.496

The fabricated samples WCE7 and WCoE7 of 90% filler and 10% epoxy resin have the higher density values among all samples with 6.609 and 6.496 g/cm^3^ respectively, while WCE1 and WCoE1 of 60% filler and 40% epoxy resin have the least density values with 2.419 and 2.409 g/cm^3^ respectively.

**Figure 5:**
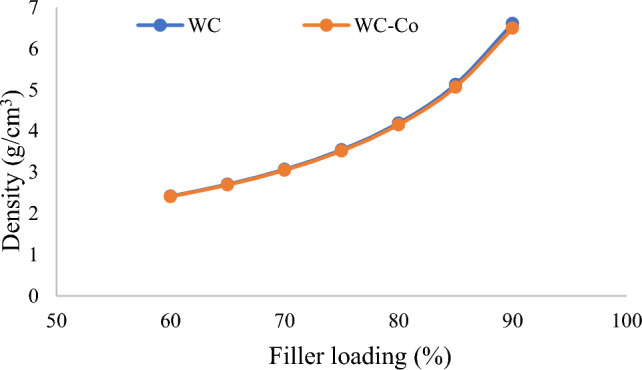
Variation of densities values of the fabricated samples with respect to filler loading.

In general, it is observed that the addition of powder to epoxy matrix increases the density of the samples as shown in Fig. 5. There are more interactions per unit length of the material in high density materials because there are more atoms and heavier atoms along the path of the photons.

### Microstructure analysis

The elemental analysis based on the quantitative EDX analysis of epoxy resin sample, tungsten carbide powder, tungsten carbide cobalt powder is listed in this section. The elemental composition of these samples by weight and atomic percentages showed the elements contained within epoxy resin, tungsten carbide, and tungsten carbide cobalt samples including carbon, oxygen, chlorine, tungsten, and cobalt as summarised in Table 8.

**Table 8:** The elemental composition by weight and atomic percentages of epoxy resin, tungsten carbide, and tungsten carbide cobalt.

Element	Epoxy resin		Tungsten carbide		Tungsten carbide cobalt	
	Weight%	Atomic%	Weight%	Atomic%	Weight%	Atomic%
C	80.75	84.92	9.46	60.56	9.69	55.84
O	18.98	14.98	–	–	1.04	4.41
Cl	0.27	0.09	–	–	–	–
W	–	–	90.53	39.43	81.53	30.66
Co	–	–	–	–	7.73	9.08
Total	100	100	100	100	100	100

The EDX analysis of tungsten carbide-based epoxy resin (WCE7) and tungsten carbide cobalt-based epoxy resin (WCoE7) composites is summarised in Table 9.

**Table 9:** The elemental composition of tungsten carbide-based epoxy (WCE7) and tungsten carbide cobalt epoxy (WC0E7) samples by weight and atomic percentages.

Element	WCE7		WCoE7	
	Weight%	Atomic%	Weight%	Atomic%
C	70.29	81.08	70.95	71.83
O	20.84	18.2	22.22	27.53
W	8.87	0.7	6.34	0.39
Co	–	–	0.22	0.05
Cl	–	–	0.24	0.09
Total	100	100	100	100

**Figure 6:**
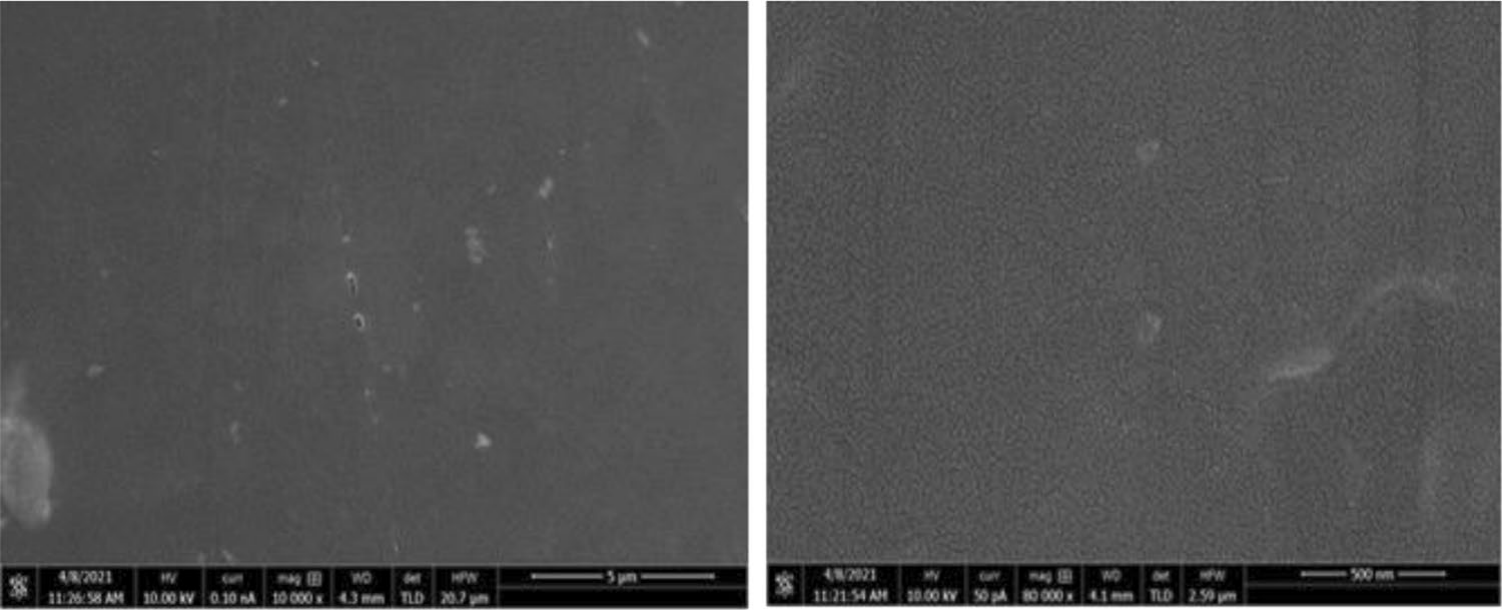
FESEM image of epoxy resin.

**Figure 7:**
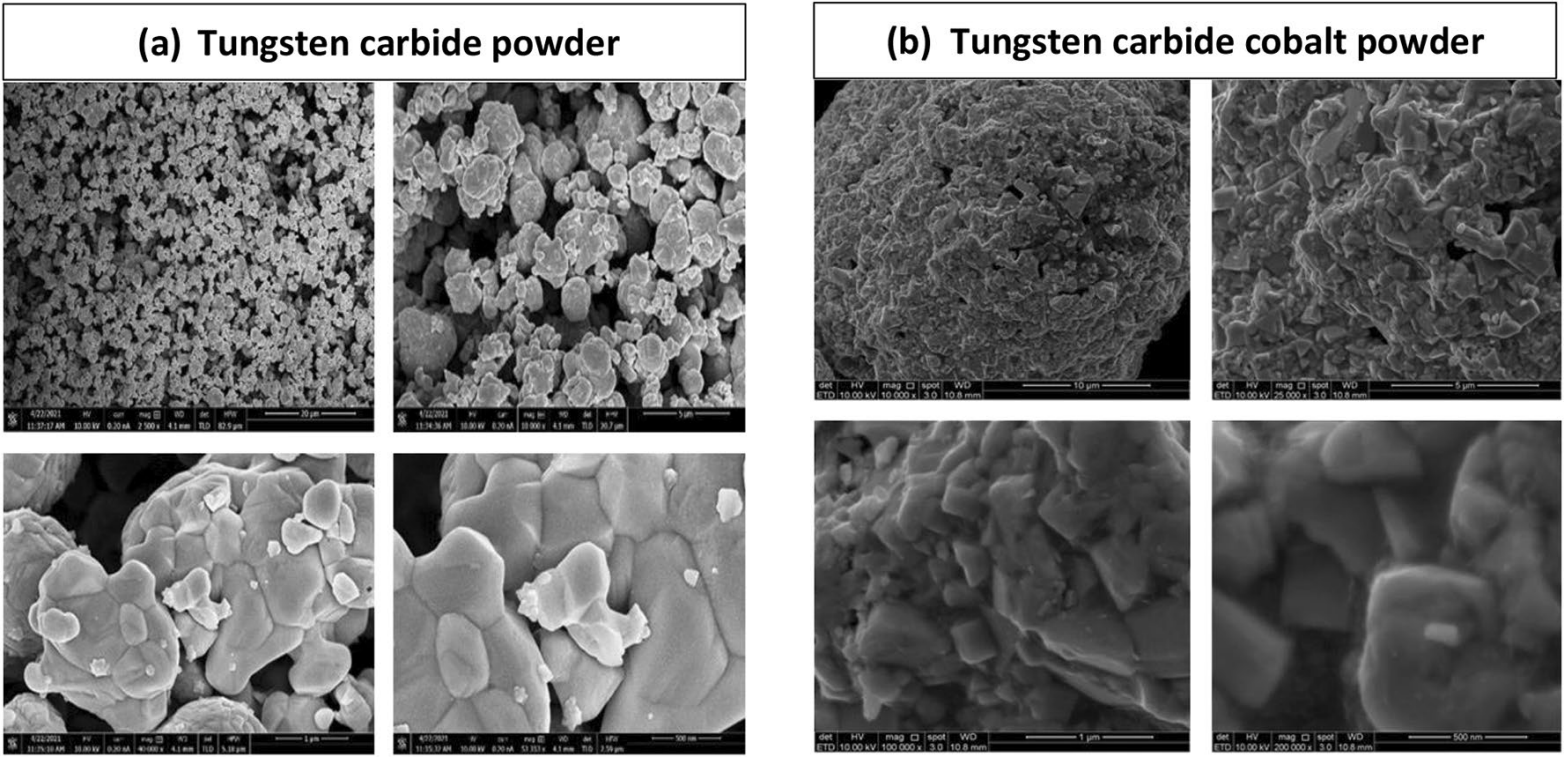
(**a**) FESEM image of Tungsten carbide powder and (**b**) FESEM image of Tungsten carbide cobalt powder.

The morphological examinations of pure powders, epoxy resin, and the composites were performed under field emission scanning electron microscope to evaluate the purity of powders and to investigate the dispersibility of powder filler with epoxy resin composition, the binding behaviour between the powder particles and polymer matrix, and the composites microstructure. One of the critical factors that contributes to composites' enhanced properties is a uniform and homogeneous distribution of fillers within the matrix. All surfaces were cleaned according to the standard procedure and coated with gold to minimise the charging effect, increase conductivity of epoxy and to enhance the images resolution. The FESEM images of epoxy resin, tungsten carbide powder, and tungsten carbide cobalt powder are illustrated in Figs. 6 and 7 respectively.

FESEM image of the morphological features of epoxy resin before incorporated with powder fillers is shown in Fig. 6. As shown in the figure, it is obvious that the fracture morphology of the neat epoxy resin is almost smooth. There were very few deformations observed in the figure.

Fracture morphology of tungsten carbide powder observed by FESEM is shown in Fig. 7a. Tungsten carbide powder particle has a hexagonal shape. A relatively uniform distribution of the tungsten carbide particles shown in the FESEM images. However, due to the small particle size and the large specific surface area that lead particles to cling together and become agglomerated, considerable agglomeration was seen under the influence of the surface energy.

FESEM image of the microstructure of tungsten carbide cobalt powder is shown in Fig. 7b. Tungsten carbide cobalt particles are polygonal in shape with an obvious grain boundary. The grey cobalt grains distributed uniformly around tungsten carbide particles as shown in Fig. 7.

**Figure 8:**
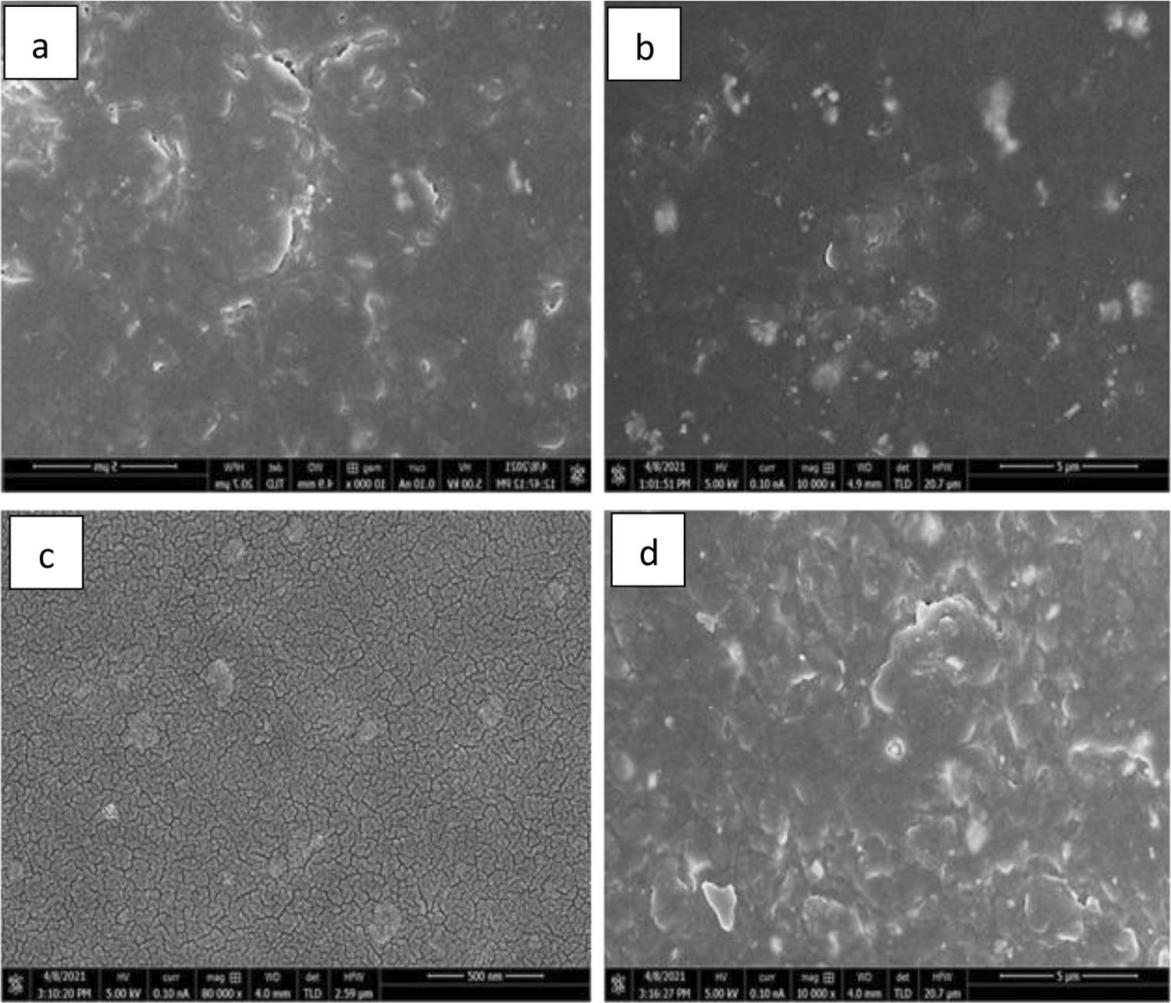
(**a**) FESEM images of the fractures surface of tungsten carbide epoxy resin composites, with 35% epoxy resin weight percentage at 5 mm resolution, (**b**) 25% epoxy resin weight percentage at 5 µm resolution, (**c**) 15% epoxy resin weight percentage at 500 nm resolution and (**d**) 10% epoxy resin weight percentage at 5 mm resolution.

One of the critical factors that contributes to composites' enhanced properties is a uniform and homogeneous distribution of fillers within the matrix. The distribution of tungsten carbide powder within the epoxy resin matrix at various weight percentages is depicted in Fig. 8.

Atomic number contrast is used to distinguish between elements with low high atomic number and high atomic number. The filler material appears to be brighter than the surrounding epoxy resin. The tungsten carbide powder filler particles dispersed well within the polymer matrix. It is obvious that the powder is dispersed evenly. However, as illustrated in Fig. 8d, number of agglomerates increases as the powder weight fraction increases.

**Figure 9:**
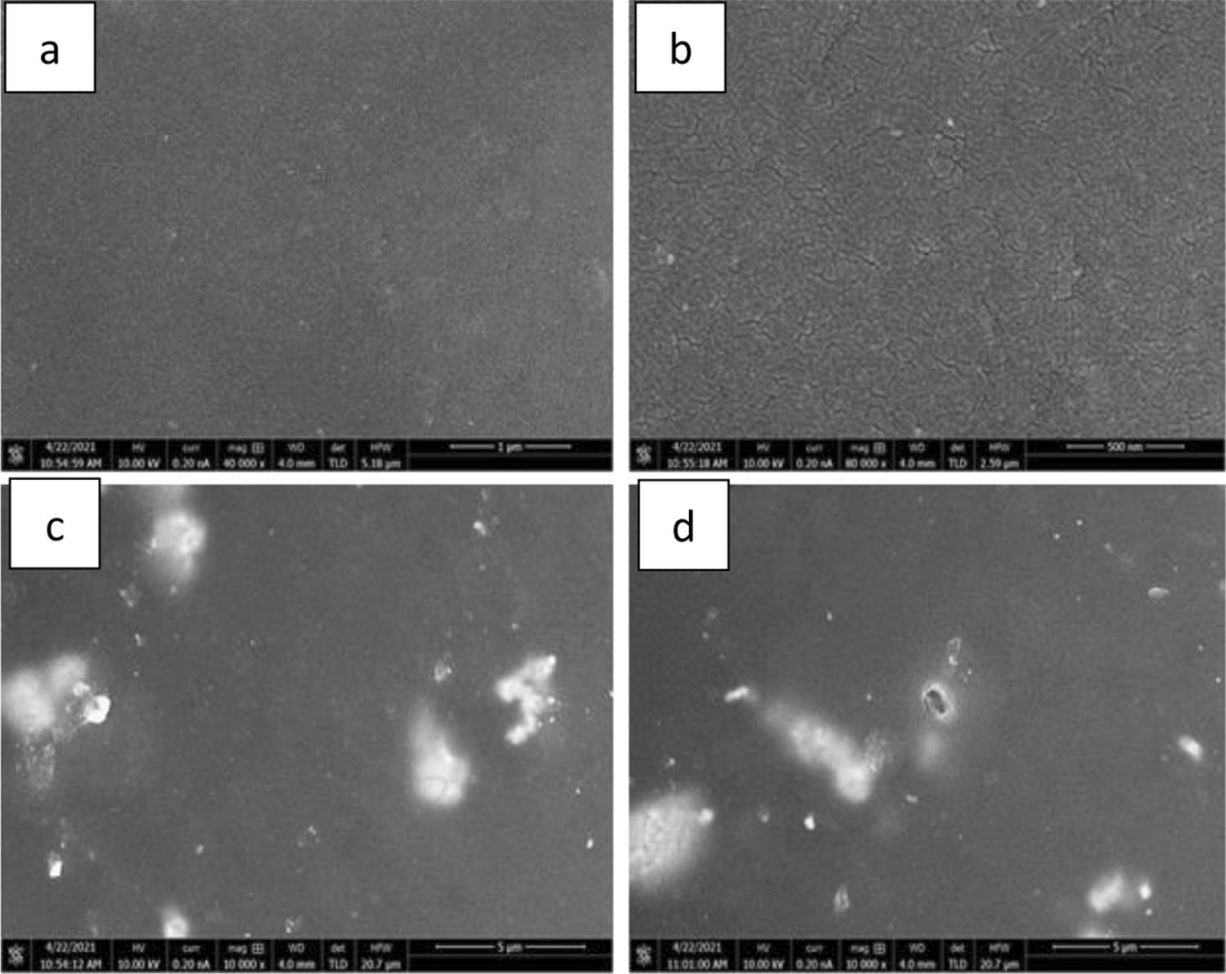
FESEM images of the fractures surface of tungsten carbide cobalt epoxy resin composites, with (**a**) 35% epoxy resin weight percentage at 1 micro, (**b**) 25% epoxy resin weight percentage at 500 nano, (**c**) 15% epoxy resin weight percentage at 5 µm and (**d**) 10% epoxy resin weight percentage at 5 µm.

FESEM images of the fracture surfaces of tungsten carbide cobalt-based epoxy resin composites are shown in Fig. 9.

FESEM images revealed a uniform and homogeneous distribution of tungsten carbide cobalt powder with no agglomeration within the polymer matrix, indicating that the samples had been successfully prepared, as illustrated in Fig. 9a,b. With a low filler loading, the fillers are seen to be uniformly dispersed in the epoxy matrix. However, as tungsten carbide cobalt particles loading increases, the dispersion of the fillers within the matrix becomes less uniform, resulting in some agglomerations with more intense white patches on the surface, as illustrated in Figs. 9 (c) and (d).

### Mechanical properties

**Figure 10:**
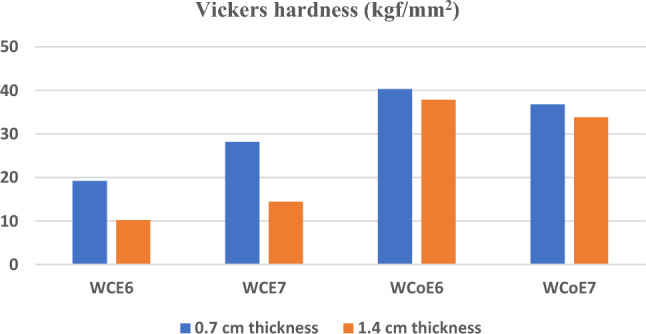
Vickers hardness values of the investigated samples with 0.7 cm and 1.4 cm thickness.

The hardness test was performed for the fabricated samples with 15% and 10% epoxy resin weight composites at two thicknesses 0.7 cm and 1.4 cm. Results of Vickers hardness test were presented in Table 10. Vickers hardness values of the fabricated samples are illustrated in Fig. 10. It is clearly observed that the average hardness values of tungsten carbide cobalt epoxy resin composite were significantly higher than the average hardness of the other composites. It was also found that the addition of tungsten carbide cobalt to the composite increased the load capacity of the epoxy resin matrix compared to other composites. This is mainly due to the superior hardness characteristics of cobalt. It is commonly known that cobalt element added to tungsten carbide to increase its hardness and toughness.

**Table 10:** The Vickers hardness values of the fabricated samples.

Sample	Thickness (cm)	Vickers hardness value (kgf/mm^2^)
WCE6	0.7	19.23
WCE6	1.4	10.23
WCE7	0.7	28.16
WCE7	1.4	14.43
WCoE6	0.7	40.30
WCoE6	1.4	37.86
WCoE7	0.7	36.76
WCoE7	1.4	33.83

Figure 10 showed that more addition of epoxy increased the hardness value, samples with 15% of epoxy resin gives higher Vickers hardness values than samples with 10% epoxy resin. It was clearly observed that increasing sample thickness from 0.7 to 1.4 cm resulted in decreasing the Vickers hardness value and this is mainly due to the arrangement of polymer chain structure that made the powder grains shifted to the centre of the sample and formation of the elastic resin layer at the surface. According to literature, it is well known that the superficial layer of the polymer sample exhibited less degree of polymerisation and lower hardness value than that of the intermediate layer (Hamouda et al.^25^; Szewczak^26^).

### Chemical properties

Fourier transform infrared spectroscopy of epoxy resin, tungsten carbide-based epoxy resin composite (WCE) and tungsten carbide cobalt-based epoxy resin composite (WCoE) was carried out to identify the chemical bonds and detect the functional groups. The FTIR spectrum of each sample was recorded in the range of 600–4000 cm^−1^.

**Figure 11:**
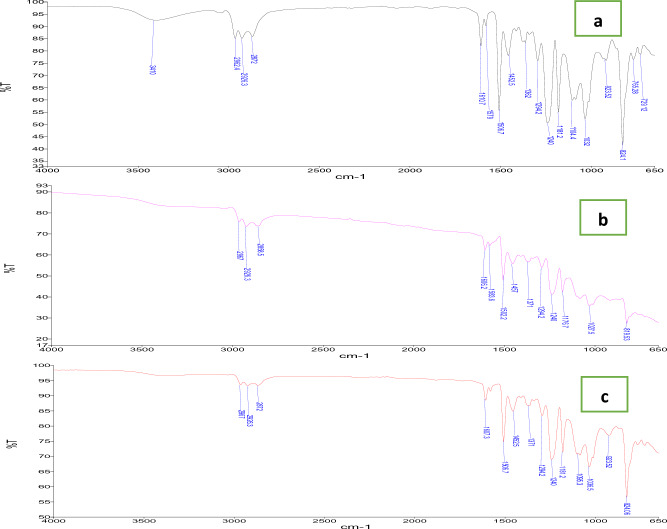
(**a**) FTIR spectrum of epoxy resin, (**b**) FTIR spectrum of WCE composite, and (**c**) FTIR spectrum of WCoE composite.

FTIR was used to investigate the interaction between the filler particles and epoxy resin polymer chain in the fabricated composites. As shown in Fig. 11 the fabricated composites have intensity change in the range of 800–1600 cm^−1^ that results in formation the linkages between epoxy and filler particles.

The infrared spectrum of epoxy resin without filler exhibits a broad peak ranging from 3200 to 3600 cm^−1^ due to O–H stretching, the sharp absorption band at 932 cm^−1^ corresponding to C–N stretching vibration^18^. Around 900 and 650 cm^−1^, FTIR spectrum of epoxy resin sample showed increased intensity with additional carbonyl ions in the polymer chain. However, addition of tungsten carbide and tungsten carbide cobalt powders to the matrix have shown less intensity changes compared to epoxy resin.

FTIR spectrum of all samples showed ether and oxy compound C–O stretch between 1362 and 1610 cm^−1^. Moreover, all spectrums shown epoxy ring at 1294 cm^−1^. C–Cl stretch observed at 1032 cm^−1^. The presence of hydroxyl groups is shown in Fig. 11c and witnessed by broad peaks. Normal polymeric OH stretch and hydroxy group H bonded, OH bonded at 3410 cm^−1^ for epoxy resin sample. The presence of unsaturated carbon bonds was confirmed by the presence of weak absorption bands at 824 and 923 cm^−1^^18^. In addition, the infrared spectrum of tungsten carbide-based epoxy resin composites showed no appreciable change in the infrared spectrum with increasing with different filler additives, which confirms that no reaction takes place between fillers and any of the components in the resin. Generally, the position of all the peaks is found to be the same in all fabricated samples. There is neither the peak shift nor a new peak or the disappearance of the peaks. There was no change of structure observed on epoxy resin matrix after adding filler powders. This indicates that the polymer filler interaction is considered a physical type of interaction not a chemical interaction.

### Mass attenuation coefficients

The fabricated samples were evaluated against gamma rays using gamma spectrometer. Narrow beam geometry was applied to measure the liner attenuation coefficients using gamma ray sources. Energy calibration of the spectrometer, the attenuation coefficient measurements, calculations of half value layer, tenth value layer, mean free path, and measurements of the radiation shielding efficiency were explained in detail in the subsection.

#### Energy calibration of the spectrometer

**Figure 12:**
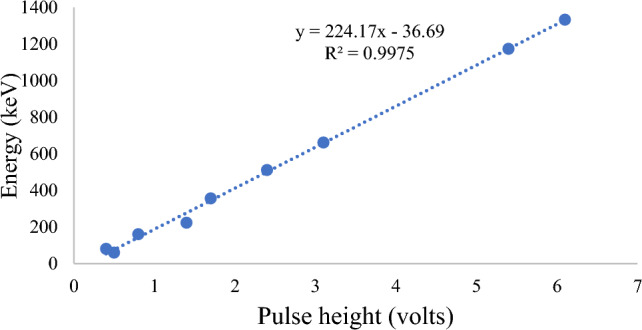
Energy calibration test for photon energy versus pulse height.

Energy calibration is the first step to perform in gamma spectroscopy experiment. This entails determining the link between the energy of a gamma ray and the counts peak produced by that energy photons. Energy calibration was performed by counting sources that emit gamma rays of known energies. In this study, the gamma radiation sources used were Am-241 (60 keV), Ba-133 (80 keV, 160 keV, 223 keV, 356 keV), Na-22 (511 keV), Cs-137 (661), Co-60 (1173.2 keV, 1332.5 keV). The energy calibration curve is the plot of gamma ray energy as a function of pulse height window as shown in Fig. 12.

#### Gamma attenuation measurements

**Figure 13:**
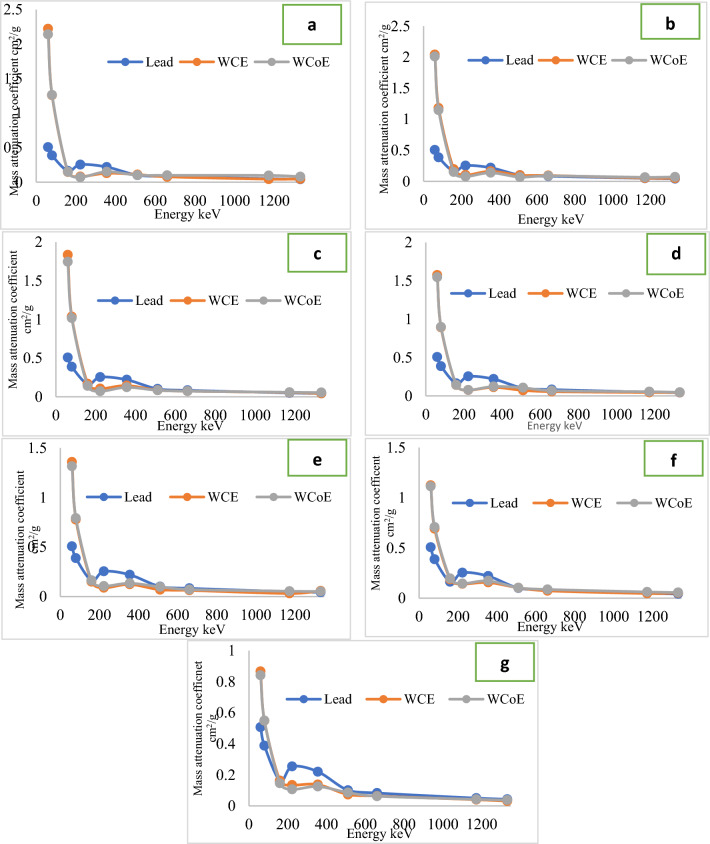
Mass attenuation coefficients of the fabricated samples respect to gamma energy, (**a**) WCE1&WCoE1, (**b**) WCE2&WCoE2, (**c**) WCE3&WCoE3, (**d**) WCE4&WCoE4, (**e**) WCE5&WCoE5, (**f**) WCE6&WCoE6 and (**g**) WCE7&WCoE7.

The linear attenuation coefficient of each composite sample was determined by the relationship between the incident and passing through gamma photons from the shielding material. The energy spectrum was used to calculate the intensity of incoming (*I*_*o*_) and passing through (*I*) gamma rays by using the number of counts under the photo peak of the spectrum. Mass attenuation coefficients of the fabricated samples were shown in Fig. 13.

High attenuation coefficient values represent a high possibility of radiation beam becoming attenuated by the fabricated composites. As radiation energy increases, the attenuation coefficient values become smaller compared to low energy regions, for both lead and the fabricated samples. The maximum separation between tungsten carbide-based epoxy resin composites and lead curves occurs at around 60 keV, which can be explained in respect to the K-absorption edge in the photoelectric absorption of radiation photons. The K-edge of lead is almost 60.6 keV, and the sudden increase in attenuation is due to the photoelectric effect of the photons.

At this energy, the difference in attenuation between the two materials is the greatest. This significant difference can be observed among all the fabricated samples with different epoxy and filler weight in compared to lead except for composites with 10% epoxy resin and 90% filler weight which made it the best combination of epoxy and filler composite in compared to lead followed by samples with 15% epoxy resin and 85% filler weight. It is clearly seen that the difference became smaller with increasing powder filler in the matrix. This discovery is supported by the fact that, when filler weight loading in the matrix rises, the atomic number and density of the composite also rise, improving the composite's radiation-shielding capabilities. Mass attenuation coefficient values of lead, tungsten carbide-based epoxy, and tungsten carbide cobalt based epoxy are 0.049 cm^2^/g, 0.043 cm^2^/g, and 0.096 cm^2^/g at 1.17 MeV and 0.042 cm^2^/g, 0.045 cm^2^/g, and 0.08 cm^2^/g at1.33 MeV respectively.

At low energy regions, the attenuation coefficient values of the composites with different filler loadings decreased sharply with increasing gamma ray energy, whereas at intermediate and high energy regions, the attenuation coefficient values were only slightly decreased with increasing gamma ray energy, and the filler loading of the composites was nearly ineffective in terms of improving the radiation attenuation performance of the investigated composites.

It can be observed that radiation attenuation results indicated the effect of composite density on shielding properties. A low-density absorber will cause less attenuation than a high-density absorber since the chances of an interaction between the radiation and the absorber's atoms are smaller. Furthermore, due to the porous nature of the material, the density determines the transmission coefficient as it pertains to the sample, as the lower the density, the higher the transmission coefficient.

The gamma ray shielding of the fabricated composites improved with an increase in the filler content. The best shielding efficiency against gamma radiation was obtained using 85% and 90% of fillers against 15% and 10% of epoxy resin respectively. Attenuation coefficient measurements results indicated that the proposed metal-polymer composites could strongly be potential candidates for gamma radiation shielding in nuclear medicine department.

#### Half value layer (HVL), Tenth value layer (TVL), and mean free path (MFP)

The half value layer and the tenth value layer are the most commonly used quantitative parameters for determining the penetrating ability of different types of radiation through different types of materials. In this study, the values of HVL, TVL and MFP were calculated for all tungsten carbide-based epoxy resin composites and tungsten carbide cobalt-based epoxy resin composites with 90% filler powder and 10% epoxy resin at 1.4 cm thicknesses for energies ranging from 60 up to 1332.5 keV as shown in Table 11.

**Table 11:** Variation of half value layer values, tenth value layer values, and mean free path values of the investigated samples as a function of energy.

Sample	Lead			WCE			WCoE		
Energy	HVL	TVL	MFP	HVL	TVL	MFP	HVL	TVL	MFP
60	0.250	0.831	0.361	0.244	0.813	0.353	0.238	0.793	0.344
80	0.266	0.885	0.384	0.330	1.097	0.476	0.331	1.100	0.477
160	0.572	1.902	0.826	0.865	2.874	1.248	0.866	2.878	1.250
223	0.247	0.821	0.356	0.867	2.881	1.251	0.897	2.981	1.295
356	0.282	0.939	0.408	0.843	2.802	1.216	0.844	2.805	1.218
511	0.563	1.873	0.813	1.564	5.198	2.257	1.501	4.989	2.166
661	0.725	2.409	1.046	1.881	6.250	2.714	1.752	5.821	2.528
1173.2	1.27	4.219	1.832	2.760	9.173	3.984	2.732	9.078	3.942
1332.5	1.301	4.325	1.878	3.079	10.23	4.443	2.999	9.964	4.327

The fabricated samples with 90% filler and 10% epoxy resin were investigated for another radiation shielding properties half value layer, tenth value layer and mean free path. Half value layer represents the thickness of fabricated samples that can exactly attenuate half of the original photon intensity. Lower half value layer indicated that smaller thickness of the sample can be used to shield incident photon intensity by half.

As seen in Table 11, the half value layer values of the investigated samples are found to be increased with the increasing of photon energy. Increasing photon energy means increasing the penetration of gamma photons through the investigated sample, which in turns increase the number of transmitted photons through the sample. Samples with higher densities have higher potential for gamma photons to strike the atoms, which increases the probability of photon interactions resulting in only a few photons being passed through the shielding material. At 60 keV, half value layer for lead, WCE, and WCoE is 0.25, 0.244, and 0.238 respectively. At higher energies up to 1.33 MeV, polymeric-based composites, while also effective at attenuating radiation, require a thicker layer to achieve efficient radiation shielding in compared to lead.

The average distance a photon can travel before interaction with the atoms of the target material known as mean free path. As shown in the table, the highest mean free path values are obtained at higher gamma energies while the lowest mean free path value obtained at lower energies. High energy gamma rays up to 1.17 and 1.33 MeV have a longer mean free path because it can traverse through a material with fewer interactions due to its shorter wavelength and higher penetration capability. Lower-energy gamma rays ranging from 60 to 661 keV, on the other hand, are more susceptible to interactions with the material, resulting in a shorter mean free path.

It can be observed that the half value layer values were found to be inversely proportional to the density of the samples. The higher density samples have the lower half value layer values, and this ascribed to the higher percentage of high atomic number elements in the samples with high density, which increased the interaction possibility between gamma radiation and the absorbing material. Thus, samples with high weight percentage of high atomic number fillers have superior shielding properties.

The values of half value layer, tenth value layer, and mean free path increase as the energy of the incident gamma photon increases from 60 to 1332.5 keV. Similar behaviour of all samples has been noticed when increasing gamma ray energy and the percentage weight of the fillers within the polymer matrix.

#### Radiation protection efficiency

**Figure 14:**
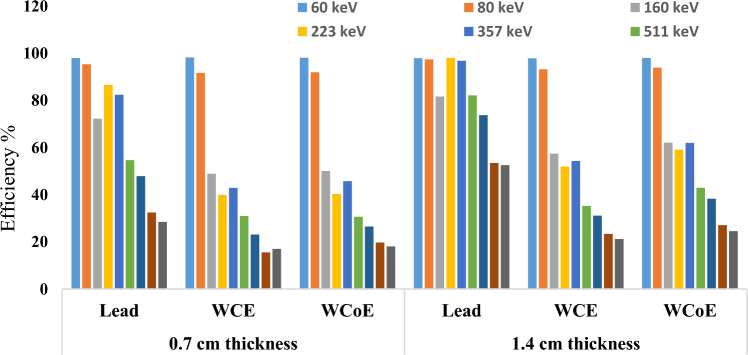
Variation of radiation shielding efficiency values for the investigated samples as function of energy at 0.7 cm and 1.4 cm thicknesses.

Radiation protection efficiency of lead and the fabricated samples with 85% filler/15% and 90% filler/10% epoxy resin at 0.7 cm and 1.4 cm thicknesses for energies ranging from 60 up to 1332.5 keV is shown in Fig. 14.

The shielding efficiencies of the investigated samples were quite close to each other. Nevertheless, the efficiencies of the composite material were better than that of lead at 60 keV. The results indicated that as the concentration of high atomic number filler in the composite increases, the probability of a gamma photon interacting with the composite material increases; thus, composites with improved gamma shielding properties are obtained. In other words, a polymer composite with a higher density and a higher atomic number concentration is more appropriate for radiation shielding applications. Furthermore, it can be observed that polymeric composites with micro-sized tungsten carbide particles and polymeric composites with nanosized tungsten carbide cobalt particles gave almost same radiation shielding efficiencies at lower gamma energies up to 223 keV, while at higher energies up to 1.33 MeV, fabricated samples with nanosized particles gave a better radiation shielding efficiencies than those with micro-sized particles, which is due to the fact that nanosized particles have a significantly higher surface area-to-volume ratio than micro-sized particles. This increased surface area allows for more opportunities for radiation interactions, including scattering and absorption. As a result, the nanosized particles can attenuate radiation more effectively than micro-sized particles. This is because radiation interacts more strongly with smaller particles due to their higher curvature and increased surface area.

These results matched the conclusions obtained by other researchers on metallic powder fillers reinforced epoxy matrix composite oxide^27,28^. The increase in filler concentration in epoxy matrix obviously increases the shielding efficiency of the resulting composite material and also increasing the mass attenuation coefficients experimentally^29,30^.

## Conclusion

Lead-free and light weighted tungsten carbide-based epoxy resin bricks were successfully fabricated and evaluated for protection against gamma radiation. In this study, tungsten carbide powder and tungsten carbide cobalt particles were observed to disperse uniformly in the epoxy resin polymer matrix. Tungsten carbide-based epoxy resin composites exhibited good gamma shielding properties especially for high filler loadings (85% and 90% filler weight percentages of total composite’s weight).

The density of the composites influenced the gamma ray shielding performance of the fabricated composites. Although increasing the composite density improved its radiation shielding performance, the density effect became ineffective at high gamma energies. The morphological properties studied for all composites as well as the powder fillers and epoxy resin. Powders used in this study were high purity powders, with a purity almost 99 to 99.5% purity percentages.

Hardness tests were performed for tungsten carbide-based epoxy resin composites to ensure the structural strength of the composites by testing the maximum load that the fabricated composites can bear. Vickers microhardness tester was used in this study for this purpose. The fabricated composites showed a good resistance, the maximum hardness was attributed to composites with small thickness. The high loading of tungsten carbide powder in the epoxy matrix improved the microhardness of the composites. Tungsten carbide-based epoxy resin composite with 10% and 15% epoxy is considered the best filler epoxy combination among all the investigated samples and gave the best shielding performance against gamma ray.

## Data Availability

Data sets generated during the current study are available from the corresponding author on reasonable request.

## References

[1] Obaid SS, Gaikwad DK, Pawar pp. (2018). Determination of gamma ray shielding parameters of rocks and concrete. Radiat. Phys. Chem..

[2] Manjunatha HC, Seenappa L, Chandrika BM, Hanumantharayappa C (2017). A study of photon interaction parameters in barium compounds. Ann. Nucl. Energy.

[3] Powell-Turner J, Antill PD, Fisher RE (2016). The United Kingdom Ministry of Defence and the European Union's electrical and electronic equipment directives. Resour. Policy.

[4] T. Union, O. Journal, and E. Union, Directive 2011/65/EU of the European parliament and of the council of 8 June 2011 on the restriction of the use of certain hazardos substances in electrical and elecronic equipment, *Off. J. Eur. Union* 1–38 (2019).

[5] European Parliament, Directive 2011/65/EU of the European Parliment and of the council on the restriction of the use of certain hazardous substances in electrical and electronic equipment (RoHS)-recast, *Off. J. Eur. Union* 88–110 (2011).

[6] AbuAlRoos NJ, Amin NA, Zainon R (2019). Conventional and new lead-free radiation shielding materials for radiation protection in nuclear medicine: A review. Radiat. Phys. Chem..

[7] Grimberg A, DiMeglio LA, Rudolph C, Rudolph A, Lister G, First L, Gershon A (2011). Mechanisms of hormone action. Rudolph’s Pediatrics.

[8] Ambika MR, Nagaiah N, Suman SK (2017). Role of bismuth oxide as a reinforcer on gamma shielding ability of unsaturated polyester based polymer composites. J. Appl. Polym. Sci..

[9] Atashi P, Rahmani S, Ahadi B, Rahmati A (2018). Efficient, flexible and lead-free composite based on room temperature vulcanizing silicone rubber/W/Bi2O3 for gamma ray shielding application. J. Mater. Sci. Mater. Electron..

[10] Chen S, Nambiar S, Li Z, Osei E, Darko J, Zheng W, Sun Z, Liu P, Yeow JT (2019). Bismuth oxide-based nanocomposite for high-energy electron radiation shielding. J. Mater. Sci..

[11] AbuAlRoos NJ, Azman MN, Amin NA, Zainon R (2020). Tungsten-based material as promising new lead-free gamma radiation shielding material in nuclear medicine. Phys. Med..

[12] Toller L, Liu C, Holmström E, Larsson T, Norgren S (2017). Investigation of cemented carbides with alternative binders after CVD coating. Int. J. Refract. Met. Hard Mater..

[13] Milman YV (2014). The effect of structural state and temperature on mechanical properties and deformation mechanisms of WC-Co hard alloy. J. Superhard Mater..

[14] Gavrish VM, Baranov GA, Chayka TV, Derbasova NM, Lvov AV, Matsuk YM (2016). Tungsten nanoparticles influence on radiation protection properties of polymers. IOP Conf. Ser. Mater. Sci. Eng..

[15] Malekie S, Hajiloo N (2017). Comparative study of micro and nano size WO3/E44 epoxy composite as gamma radiation shielding using MCNP and experiment. Chin. Physi. Lett..

[16] Noor Azman NZ, Siddiqui SA, Low IM (2013). Synthesis and characterization of epoxy composites filled with Pb, Bi or W compound for shielding of diagnostic x-rays. Appl. Phys. A.

[17] Dong M, Xue X, Yang H, Liu D, Wang C, Li Z (2016). A novel comprehensive utilization of vanadium slag: As gamma ray shielding material. J. Hazard. Mater..

[18] Joshi S, Snehalatha V, Sivasubramanian K, Ponraju D, Jayaraman V, Venkatraman B (2019). Radiation stability of epoxy-based gamma shielding material. J. Mater. Eng. Perform..

[19] Abualroos NJ, Zainon R (2021). Fabrication of new non-hazardous tungsten carbide epoxy resin bricks for low energy gamma shielding in nuclear medicine. J. Phys. Commun..

[20] Giménez MAN, Lopasso EM (2018). Tungsten carbide compact primary shielding for small medium reactor. Ann. Nucl. Energy.

[21] Chang L, Zhang Y, Liu Y, Fang J, Luan W, Yang X, Zhang W (2015). Preparation and characterization of tungsten/epoxy composites for γ-rays radiation shielding. Nucl. Instrum. Methods Phys. Res. Sect. B Beam Interact. Mater. Atoms.

[22] Aygün B (2019). Epoxy based metal and metal oxide doped new composite neutron and gamma radiation moderator material. Erzincan Üniversitesi Fen Bilimleri Enstitüsü Dergisi.

[23] Harish V, Nagaiah N, Prabhu TN, Varughese KT (2009). Preparation and characterization of lead monoxide filled unsaturated polyester based polymer composites for gamma radiation shielding applications. J. Appl. Polym. Sci..

[24] Hynes A, Scott DA, Man A, Singer DL, Sowa MG, Liu KZ (2005). Molecular mapping of periodontal tissues using infrared microspectroscopy. BMC Med. Imaging.

[25] Hamouda IM, Abudllah M, Almalki M (2020). Effect and correlation of testing load and specimen’s thickness on the hardness and percent depth of cure of condensable composite resins. Dent. Oral Maxillofac Res..

[26] Szewczak A, Szeląg M (2020). Physico-mechanical and rheological properties of epoxy adhesives modified by microsilica and sonication process. Mater..

[27] Abdalsalam AH, Şakar E, Kaky KM, Mhareb MH, Şakar BC, Sayyed MI, Gürol A (2020). Investigation of gamma ray attenuation features of bismuth oxide nano powder reinforced high-density polyethylene matrix composites. Radiat. Phys. Chem..

[28] Mirji R, Lobo B (2020). Study of polycarbonate–bismuth nitrate composite for shielding against gamma radiation. J. Radioanalyt. Nucl. Chem..

[29] Atef S, El-Nashar DE, Ashour AH, El-Fiki S, El-Kameesy SU, Medhat M (2020). Effect of gamma irradiation and lead content on the physical and shielding properties of PVC/NBR polymer blends. Polym. Bull..

[30] Li R, Gu Y, Zhang G, Yang Z, Li M, Zhang Z (2017). Radiation shielding property of structural polymer composite: Continuous basalt fiber reinforced epoxy matrix composite containing erbium oxide. Compos. Sci. Technol..

